# Supplementary material for: Subsurface fracturing of sedimentary stones caused by bullet impacts
Source: PLoS One. 2023 Oct 25;18(10):e0292351. doi: 10.1371/journal.pone.0292351 (PMC10599578; doi:10.1371/journal.pone.0292351)
Supplement: S1 Appendix — (DOCX) [file pone.0292351.s001.docx]

APPENDIX 1: Uncertainty Calculations

Uncertainty in the distance from the crater centre measurements is estimated to be ± 2 mm based on the contribution of several factors: (i) The measurement of section locations during the cutting process. (ii) The possible loss of material at the edges of thin sections during production, though every effort was made to minimise this. (iii) Geo-referencing of thin section scans and photo-mosaics. Digitisation in QGIS was the primary source of uncertainties in the calculation of fracture intensities. The optical thin-section scans used for digitisation are limited in their resolution, despite a very high resolution of scanning (6400 dpi). Fracture apertures narrow to the point they become indistinguishable from the pixelation of adjacent grain boundaries. Following the method of Campbell et al. (2021), fracture traces were terminated if there was no distinguishable aperture when a fracture joined a grain boundary, or there was no clear continuation of the fracture (open or closed) beyond that grain (Figure A1a).

Fracture walls or grain boundaries appear pixelated at high magnifications, so fractures were digitised conservatively as a minimum extent, i.e. polylines were terminated at the last pixel identifiable as part of the fracture (Figure A1b). This uncertainty (pixelation distance) was individually measured for each sample, with values of 0.031 mm for SRS_09 sections and 0.026 mm for CHCL_09 sections. The length of fractures was also extended at all isolated tips (i.e. ‘I’ nodes) by the measured distance to calculate an uncertainty for fracture topology parameters. For each point of the sample grid there is therefore a minimum and maximum calculated fracture intensity value.


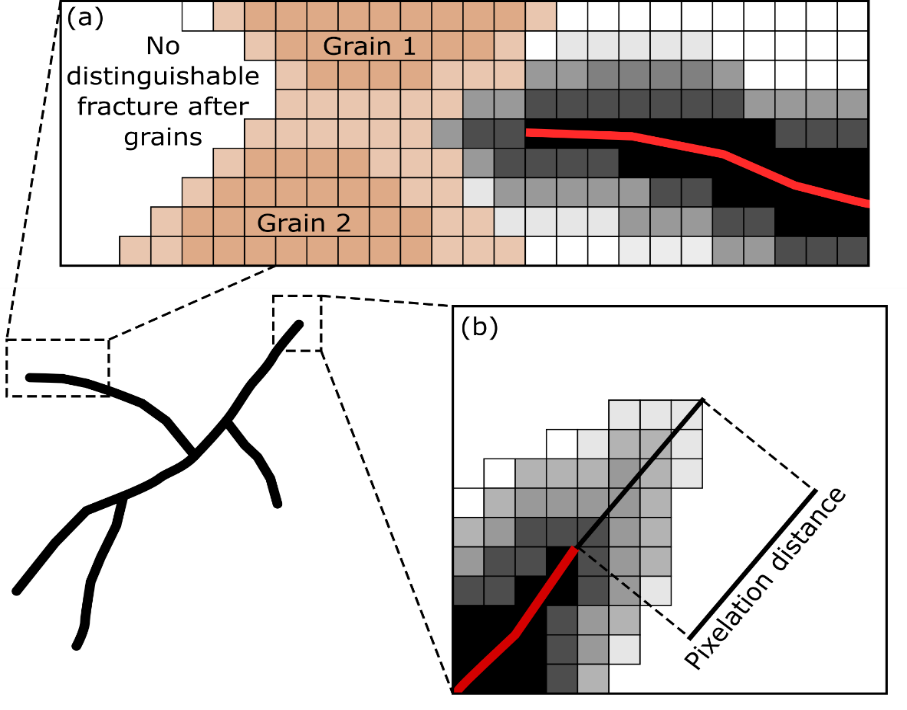


Figure A1: (a) Diagram showing how digitised fracture traces were terminated when there was no distinguishable fracture aperture after it reached grain boundaries. (b) Diagram showing how fracture tips (‘I’ nodes) are pixelated instead of being a sharp boundary. Fractures were digitised conservatively, terminating at the last pixel identifiable as the fracture (red line). The pixelation of the fracture boundary was measured for each thin section and taken as the uncertainty when calculating fracture topology parameters.

*Sandstone target*: The largest negative and positive uncertainties for *P_21_* values are - 0.0101 mm^-1^ and + 0.0720 mm^-1^ respectively, with an average uncertainty of + 0.0004 mm^-1^.

*Limestone Target*: The largest negative and positive uncertainties for P_21_ values are - 0.0178 mm^-1^ and + 0.018 mm^-1^ respectively, with an average uncertainty of + 0.0005 mm^-1^.
