# Supplementary material for: Subsurface fracturing of sedimentary stones caused by bullet impacts
Source: PLoS One. 2023 Oct 25;18(10):e0292351. doi: 10.1371/journal.pone.0292351 (PMC10599578; doi:10.1371/journal.pone.0292351)
Supplement: Attachment — Submitted filename: PONE-D-22-33228 Review Report.docx [file pone.0292351.s004.docx]

**Review Report**

The research article titled “**Multilayer Bio composites of PLA Woven Fabric and PBS Sheets Using Compression Molding Process**” is based on the development of composite sheets using PLA and PBS.

It is suggested to the author to do the extensive literature review to do research on these types of composites made from PLA. There are a lot of technical and grammatical mistakes in this manuscript.

The author has no basis knowledge about multilayer woven fabrics and multilayer composites.

Please study the basis terminologies about weaving and fabrication of composites.

1. Page 1 line 34, “The relationship between the design and construction of biocomposites structure and their properties is an important factor to the composite application.” What is the difference between design and structure.
2. “In this study, the biocomposites of poly(butylene succinate) (PBS) reinforced with poly(lactic acid) (PLA) woven fabric to improve mechanical properties of PBS matrix was fabricated by compression molding process.” No sentence structures.
3. Page 1 line 47, The impact and peel strength of PLA twill 2/2 weave biocomposite are higher than the plain weaving.” The author doesn’t have the basic knowledge about the field.
4. Page 3 line 5, “The main objective of research is to compare the weave pattern and number of layers of composites fabricated from PLA woven fabric reinforcement.
5. Page 3 line 27, “Poly(lactic acid) yarn was woven with auto-weaving machine (CCI, SL8900 evergreen model, Taiwan).” How the yarn was directly woven? Why the complete process is not mentioned?
6. Page 3 line 29 “The weaving machine setting comprised of weft frequency 50 threads/inch, weft frequency 50 threads/inch, fabric width 16 inches.”
7. This study can be report but not a technical research publication.
8. The English writing is very poor.
9. The references are with incomplete details.

I found no new findings in this research article.

This article has a lot of language errors, and no proper scientific presentation is followed.

This work can be report but not the novel idea or any new addition in previous practices, so it is **strongly Rejected for publication** in the journal. The language and writing style is so unethical according to research norms.
