# Supplementary material for: Subsurface fracturing of sedimentary stones caused by bullet impacts
Source: PLoS One. 2023 Oct 25;18(10):e0292351. doi: 10.1371/journal.pone.0292351 (PMC10599578; doi:10.1371/journal.pone.0292351)
Supplement: Attachment — Submitted filename: Response_to_Reviewers.docx [file pone.0292351.s005.docx]

**Reviewer #1**

The MS examines the effects of bullet damage on building stones. The paper is clearly written and well illustrated, and the topic is likely of non-trivial interest. The fracture analysis seems to be correct.

The work is primary scientific research. The experiments are appropriate and well explained. The fracture analysis is excellent. The conclusions are supported by the data and the discussion.

76 The strength of sandstones and limestones varies a lot with compaction, natural cement, and porosity. This is true even amongst building stones. To help make these results more widely transferable In addition to the mechanical properties that are provided I suggest providing a bit more information about the petrography (cements, porosity) of these two rock types. I see some porosity values in line 252. Did I miss something earlier?

- The porosity of each lithology is provided in the material descriptions (Lines 73 and 78).
- The cement type for each lithology has been added into the lithology descriptions on line 73 ‘With a porosity of 11% and intergranular cement comprising quartz overgrowths, it is generally massive, with some target blocks exhibiting visible beds of coarser grains (∼1 mm) (Figure 2a).’ and line 79 ‘The majority of intergranular cement comprises sparry calcite, though areas of smaller grain sizes tend towards microcrystalline calcite cement.’

82 ‘Inherent’ fractures seems cryptic. Maybe this is a term or art in building stones? Does this mean ‘no pre-existing’ fractures? Does this include naturally occurring microfractures? Some building stones have strength anisotropy due to microfracture arrays. These can be diagnosed (Anders et al. 2014, J. Str. Geol.)

- The term inherent has been replaced throughout with ‘pre-existing’ for clarity.

105 Were any precautions like pre-sectioning application of epoxy taken to minimize or eliminate sample preparation damage? (see also 144)

- Samples were impregnated with epoxy resin prior to sectioning in order to minimise additional damage. Explanation of this has been added in line 108 ‘The target block was impregnated with epoxy resin prior to sectioning in order to minimise further damage to the samples.’
- Further mention of this has been added to line 119 ‘Some thin sections were subject to material loss during section production, though every effort was made to prevent this with the pre-sectioning application of epoxy resin.’

112 What do you mean by ‘closed fractures’? Does this mean that no open space was visible at the magnifications used? Or are these somehow otherwise closed? Later you use the term ‘open extensional fractures’, so could ‘closed fractures’ signify a different fracture mode? Probably best to clearly define what you mean (categories) at the outset.

- The term closed fractures is defined on line 117.

152 Interesting texture. Is this a very porous sandstone?

- The sandstone is not particularly porous, with an average porosity of @11%.

160 Since such ‘within-grain’ fractures are naturally occurring in many sandstones (Anders et al. 2014) do you have samples from distant from the impact sites to corroborate that some of these are not part of a naturally occurring population?

- Samples from undamaged blocks of the same lithology were used to corroborate that these fractures are impact induced. This is explained on Line 83.

Fig. a, b. Although I suppose the scale is obvious, it would be a good idea to put graphic bar scales on the images. Check other diagrams for scales.

- The images used in Figure 1a,b were not taken by the authors and no information with regards to scale is available from the source of the images.
- All other figures contain scale bars where appropriate.

**Reviewer #2**

Reviewer #2: Review Report
The research article titled “Subsurface fracturing of sedimentary stones caused by bullet impacts” investigates the subsurface damage caused by bullet impacts, which is not apparent from surface inspection, in order to better understand the geometry and mechanics of this form of conflict damage to heritage.

It is suggested to the author to do the extensive literature review to do research on these types of damages.

The author has no basis knowledge about multilayer woven fabrics and multilayer composites.

Please study the basis terminologies about the construction field.

1. This study could be a report for only one building, but as the bullet is concerned, there are numerous structures and material for building construction so this study could not be helpful to draw any conclusion for benefit at large scale.

2. This study is focused on the political issues rather than scientific problems, so it could not be accepted as a research publication. Figure 1.

3. There is no specific and valuable objective of this study.

4. The references are with incomplete details.

I found no new findings in this research article.

This article has a lot of language errors, and no proper scientific presentation is followed.

This work can be report but not the novel idea or any new addition in previous practices, so it is Rejected for publication in the journal.

The language and writing style is so unethical according to research norms.

The attached review by Reviewer #2 was for a paper titled ‘Multilayer Bio composites of PLA Woven Fabric and PBS Sheets Using Compression Molding Process’.

As such, no response can be provided to the comments of Reviewer #2.
