# Supplementary material for: Subsurface fracturing of sedimentary stones caused by bullet impacts
Source: PLoS One. 2023 Oct 25;18(10):e0292351. doi: 10.1371/journal.pone.0292351 (PMC10599578; doi:10.1371/journal.pone.0292351)
Supplement: Attachment — Submitted filename: Comments on the manuscript.docx [file pone.0292351.s006.docx]

The research paper investigates the impact of bullet damage on building stones. It is evident that the paper is well-written, with clear explanations and effective illustrations. The topic itself is likely to generate significant interest. The fracture analysis conducted in the study appears to be accurate and well-executed.

This work represents original scientific research, and the experiments conducted are appropriate and thoroughly described. The fracture analysis conducted in the study is highly commendable. The conclusions drawn from the data and the subsequent discussion are well-supported and align with the findings.

1. Abstract: Line 10- Although impacts from small arms and shrapnel leave relatively inconspicuous impact scars, they may elevate the risk of future stone deterioration. How it effects the stone deterioration any literature work (may word is used means not clear).
2. Microstructural analysis of subsurface damage using thin section: It is suggested to evaluate subsurface damage using CT-Scan (or other nondestructive methods), which is a better approach than the thin section because in thin, the actual subsurface properties will be disturbed during sample preparation.
3. The literature part is very small and needs to extend this part.
4. The literature about stone deterioration is not added to the literature part.
5. Some results part is discussed in the materials and method section, which should be moved to the results and discussion section (for example, line NO. 83-85).
6. How many sandstone and limestone samples are tested? Not mentioned in the materials and methods section.
7. It is better to evaluate the bullet impact from different angles and distances.
8. In figure 2 the author stated that there was no pre-existing fracturing in the undamaged section. First the author did not show the map as shown for fractures in Figure 4. Where one can compare the results. Furthermore, the author did not take micrographs on the same scale.
9. The unit of P-wave velocity is not added.
10. It is suggested to add results about the mineralogy of these sandstone and limestone samples. Did mineralogy impact the fracture propagation or type of fracture?
11. In Table 2 the units are not added.
