# Supplementary material for: Subsurface fracturing of sedimentary stones caused by bullet impacts
Source: PLoS One. 2023 Oct 25;18(10):e0292351. doi: 10.1371/journal.pone.0292351 (PMC10599578; doi:10.1371/journal.pone.0292351)
Supplement: Attachment — Submitted filename: PLOS One Response to reviewers.docx [file pone.0292351.s007.docx]

**Reviewer 3:**

This revised manuscript investigated the bullet impacts induced subsurface damage of two sedimentary rocks of sandstone and limestone through controlled firearm experiments. The crater, the radial fractures and spallation and the subsurface damage below the impacts were observed. The paper is clearly written and well illustrated. This manuscript has some novelty on the investigation of heritage building materials and the research is meaningful for heritage conservation. My particular comments are as follows.

1. Initial damage evaluation: Basic physical and mechanical properties of these two rocks are given but the initial damage evaluation before the impact is not given. This is important to the further damage and crack formation.

- The pre-impact state of the rock has been evaluated by examining samples that were not subjected to bullet impact (Lines 83-85). These revealed there was no initial damage. This point is elaborated in the reply to review #4, point 8 below.

2. The effects of impact velocity and mass: A high-speed impact induced damage heavily depends the physical and mechanical properties of the rock, it also depends on the impact mass and impact velocity. These effects should be discussed.

- Table 2 and the discussion from lines 215 to 338 deal with the effect of impact velocity. As outlined in point 7 above, the number of impact variables was minimised: there is lot of new material to report from these two experiments alone.

3. This is a scientific research on the bullet impact induced damage evaluation. It is not necessary to introduce some non-scientific issues or statement. On this sense, Lines 29-35 in the Introduction are not appropriate and suitable.

- We suggest that these lines are appropriate because they are the motivation for paper and provide a compelling contemporary context; however, if the editor requests, we can remove them.

**Reviewer 4:**

The research paper investigates the impact of bullet damage on building stones. It is evident that the paper is well-written, with clear explanations and effective illustrations. The topic itself is likely to generate significant interest. The fracture analysis conducted in the study appears to be accurate and well-executed.

This work represents original scientific research, and the experiments conducted are appropriate and thoroughly described. The fracture analysis conducted in the study is highly commendable. The conclusions drawn from the data and the subsequent discussion are well-supported and align with the findings.

1. Abstract: Line 10- Although impacts from small arms and shrapnel leave relatively inconspicuous impact scars, they may elevate the risk of future stone deterioration. How it effects the stone deterioration any literature work (may word is used means not clear).

- The word ‘may’ has been removed from this sentence in the abstract to avoid unnecessary ambiguity. Paragraph 2 and 3 of the introduction discuss literature on bullet and shrapnel impacts into stone, and how damage can influence future deterioration

2. Microstructural analysis of subsurface damage using thin section: It is suggested to evaluate subsurface damage using CT-Scan (or other nondestructive methods), which is a better approach than the thin section because in thin, the actual subsurface properties will be disturbed during sample preparation.

- The authors took every precaution during the preparation of thin sections to minimise alterations and disturbance of subsurface features. This is addressed in Line 108 ‘The target block was impregnated with epoxy resin in order to minimise further damage to the sample’
- The authors are aware of the use of CT scanning for subsurface observations. We conducted test scans of the target block but found that the size of sample blocks precluded the use of such methods to analyse fractures at the same scale as thin sections. Target blocks would have needed to be subsampled to obtain the suitable resolution, introducing the same risk of additional damage as thin sections.

3. The literature part is very small and needs to extend this part.

- The reason for the small literature part is that research on this topic is in fact very limited (which is one reason why this study is significant). The 13 references cited are the most important previous literature. We have emphasized this by changing lines 36-37 to:

‘There are very few detailed studies of the surface damage caused by bullet and shrapnel impacts, and its relationships to the subsurface.’

4. The literature about stone deterioration is not added to the literature part.

- Lines 52-61 in the literature part specifically address deterioration, and the 7 references cited are the critical ones for the study. If the reviewer has any other specific suggestions, we would be glad to consider them.

5. Some results part is discussed in the materials and method section, which should be moved to the results and discussion section (for example, line NO. 83-85).

- The interpretation has been removed from this line, which now reads ‘Thin section micrographs from undamaged samples of each lithology show no pre-existing fractures (Figure 2). We can therefore be confident that all damage reported is the result of bullet impacts and not inherited.’

6. How many sandstone and limestone samples are tested? Not mentioned in the materials and methods section.

- Clarification of the number of samples used for the fracture analysis added to line 107: ‘Polished thin sections were cut from one damaged sample of SRS and one damaged sample of CHCL…’

7. It is better to evaluate the bullet impact from different angles and distances.

- The purpose of this study was to investigate and compare the subsurface damage between different stone types as a baseline for future comparisons. In order to do so, the number of impact variables (e.g. lithology, angle, distance) were minimised.
- Previous investigations published by the authors [16, 24] evaluated the effects of the angle of impact. They also suggest that target material has a considerable control on the resulting damage, a conclusion supported by the results of this study.
- The effects of engagement distance are the subject of ongoing investigation.

8. In figure 2 the author stated that there was no pre-existing fracturing in the undamaged section. First the author did not show the map as shown for fractures in Figure 4. Where one can compare the results. Furthermore, the author did not take micrographs on the same scale.

- The reviewer may not appreciate that it is impossible to use an invasive technique such as thin section analysis to examine the undamaged sample. Nevertheless, following the response to point 5 above, it is clear that the damage was created by the impacts.
- Micrographs were all taken at the same scale.

9. The unit of P-wave velocity is not added.

- The unit of P-wave velocity has been added to the header in Table 2. The values in Line 208 are the ratio of two velocities and are unit-less.

10. It is suggested to add results about the mineralogy of these sandstone and limestone samples. Did mineralogy impact the fracture propagation or type of fracture?

- The mineralogy of the samples is described in lines 70-88. Extra detail has been added. Mineralogy did impact fracture propagation. This is described in the results section which contrast the sandstone with the limestone targets, and the discussion deals with the mechanical reasons for the different responses in lines 226-259. The impacts of the mineralogy have been summarized in Figure 9.

11. In Table 2 the units are not added.

- The units for strain rate are in the column header (s^-1^)
- The ratio of impact velocity to P-wave velocity is unit-less. The unit of each has been added to the column header for clarity.
